# Hibiscus flower extract selectively induces apoptosis in breast cancer cells and positively interacts with common chemotherapeutics

**DOI:** 10.1186/s12906-019-2505-9

**Published:** 2019-05-06

**Authors:** Christopher Nguyen, Kiruthika Baskaran, Alaina Pupulin, Ivan Ruvinov, Ola Zaitoon, Sahibjot Grewal, Benjamin Scaria, Ali Mehaidli, Caleb Vegh, Siyaram Pandey

**Affiliations:** Department of Chemistry and Biochemistry, University of Windsor, 401 Sunset Ave, Windsor, ON N9B 3P4 Canada

**Keywords:** Hibiscus, Breast cancer, Chemotherapeutic interactions, Natural health products, Adjuvant therapy, Apoptosis, Taxol, Cisplatin, Tamoxifen

## Abstract

**Background:**

Current therapeutic approaches to treat metastatic breast cancer, although effective, have shown many inadvertent side effects such as genotoxicity due to a lack of selectivity. Thus, these treatment plans are not suitable for long-term usage. Natural health product extracts are safe for long-term consumption and some have shown to be medicinally active containing multiple bioactive compounds able target multiple vulnerabilities in cancer. One of which, *Hibiscus rosa-sinesis* (hibiscus) extract, has been reported to have many medicinal and anticancer properties due to its antioxidant and hypolipidemic effects. However, its efficacy against breast cancer has not been fully investigated and characterized. If effective against cancer, hibiscus extract could potentially be combined with chemotherapeutic treatments in adjuvant therapy to reduce chemotherapy-inducing side effects.

**Method:**

We have investigated aqueous hibiscus flower extract anticancer efficacy, selectivity, and interactions with chemotherapeutics taxol, cisplatin, and tamoxifen in estrogen-receptor positive breast cancer cells, triple-negative human breast cancer cells, and normal non-cancerous cells. Apoptotic morphology and biochemical marker expression were assessed to determine the extent anticancer efficacy of hibiscus. Mitochondrial membrane potential reduction and reactive oxygen species generation were quantified using fluorogenic dyes to determine the mechanism of hibiscus extract action.

**Results:**

Hibiscus extract was able to selectively induce apoptosis in both triple-negative and estrogen-receptor positive breast cancer cells in a dosage-dependent manner. Most importantly, addition of hibiscus extract was found to enhance the induction of apoptosis of chemotherapy treatments (taxol and cisplatin) in triple-negative breast cancer cells when compared to treatment alone. Moreover, hibiscus extract addition to chemotherapy treatment was able to increase oxidative stress and decrease mitochondrial membrane potential compared to individual treatments.

**Conclusion:**

Hibiscus extract is effective on breast cancer, most notably on generally resistant triple-negative breast cancer, while being selective for normal healthy cells. Hibiscus extract could supplement chemotherapeutic regimens as an adjuvant and lead to a more efficacious treatment approach to reduce chemotherapy dosages and related toxicity.

## Background

Breast cancer is the most prevalent cancer among women worldwide, accounting for 25% of cancer incidence and 15% of cancer deaths among females [1]. Current work has developed and enhanced prediction models, screening methods, diagnostic tools, and disease management [2–6]. However, breast cancer treatment approaches become more complicated once the disease progresses to the complex metastatic stage. Although surgery to remove tumours in breast cancer has a high probability of survival, the majority of breast cancer related deaths are not from the primary tumour itself, but a result of metastasis to organs [7].

Apoptosis is the complex and ordered physiological process of cell death. An understanding of cell death, particularly in relation to cancer, allows for an assessment of the pathogenesis and treatment of the disease [8]. The exploitation of cellular vulnerabilities in cancerous cells, including oxidative stress and mitochondrial membrane destabilization, by therapeutic agents could trigger apoptosis and potentially eradicate the disease [9, 10]. Indeed, most therapeutics have been developed to induce cell death. However, many treatments are unfortunately nonspecific for cancer and can additionally target healthy non-cancerous cells eventually leading to inadvertent side effects and toxicity [11, 12].

Current treatments for metastatic breast cancer include adjuvant chemotherapy using cytotoxic drugs including anthracyclines, taxane-based, and platinum-based drugs [13]. Although both taxane-based and platinum-based chemotherapeutics have shown effectiveness in treating breast cancer, both drugs have exhibited toxicity and lack of selectivity to support a long-term treatment plan [11, 12]. One study evaluating over 1000 patients found that treatments of anthracycline and taxane-based adjuvant strategies led to a higher pathologic complete response and higher survivability. However, a high risk of tumour relapse is possible if the tumour is not completely eradicated [14, 15]. Thus, there exists a great need for a treatment that can avoid toxicity in treatments while also able to be used on a long-term basis.

Natural health products (NHPs) are materials isolated from various food and plant sources that have been shown to have medicinal properties [16]. The commonly used chemotherapeutic taxol was isolated from the bark extract of the Pacific yew tree, *Taxus brevifolia*, when the extract was shown to have a cytotoxic effect [17]. Although many treatments have been derived from natural sources, we have yet to exhaust nature’s vast variety of selection. It is possible that a well-tolerated, highly potent anticancer compound is still left to be discovered and developed into a novel cancer therapeutic. Indeed, many NHPs have been shown to induce apoptosis selectively in cancer cells, including our research into dandelion root, lemongrass, and long pepper extracts [18–20]. Traditionally, NHPs have been used widely as both medicinal and food products [21].

Hibiscus flower (*Hibiscus rosa-sinesis)* has traditionally been used and has been shown to have high pharmacological potential to treat disorders such as hypertension and pyrexia [22]. Further, hibiscus extract (HE) has been shown to have significant antioxidant and hypolipidemic effects [23]. Previous work on hibiscus has indicated that HE exhibits significant anticancer efficacy on prostate cancer, leukemia, gastric cancer, and human squamous cell carcinoma [24–27]. A previous study of *Hibiscus syriacus* observed that several triterpenoids from HE were able to inhibit triple-negative breast cancer cell viability with limited toxicity on normal cells [28]. This work lends support to the notion that a whole plant extract of hibiscus could contain anticancer compounds while being well-tolerated.

Triple-negative breast cancer accounts for approximately 15–20% of all breast cancers and is characterized by negative expression of estrogen and progesterone receptors as well as HER2 protein [29]. Many challenges arise in the treatment of triple-negative breast cancer due to poor prognosis resulting from the lack of actionable targets in order to use a specific targeted therapy able to combat the disease [30, 31]. As such, the discovery and development of therapies able to target triple-negative breast cancer is of great importance.

We aimed to investigate the efficacy of HE against breast cancer by assessing the toxicity of HE treatment on human triple-negative and estrogen-receptor positive (ER+) breast cancer cells. Further, we aimed to investigate its interaction with current chemotherapies to assess the potential of its use in adjuvant therapies.

In this study, we have shown that aqueous HE is able to induce apoptosis in breast cancer cell models in vitro in a dose-dependent manner. We have also shown that HE treatment shows selectivity for cancer cells, with minimal effect on normal non-cancerous cells. Most importantly, we wanted to investigate the potential of using HE as an adjuvant to current chemotherapeutic treatments. We have demonstrated HE treatments (when combined with chemotherapeutic treatments) enhanced the induction of apoptosis when compared to individual treatment alone. These results support the possibility of supplementing chemotherapeutic regimens with HE, which has shown to be well-tolerated in normal non-cancerous cells. This may lead to a better combined effect, reducing the chemotherapeutic dosages and related toxicity.

## Methods

### Hibiscus leaf aqueous extraction

Hibiscus flower (*Hibiscus rosa-sinensis*) were obtained from Premier Herbal Inc. (Toronto, ON, Canada). This aqueous extraction protocol is similar to that previously published with the following modifications [18, 19]. The flowers were grinded using a coffee grinder into a fine powder. The powder was extracted in boiled double distilled water (ddH_2_O) (1 g leaf powder to 10 mL ddH_2_O) at 60 °C for 3 h. The extract was then run through a cheese cloth and then filtered via gravity filtration with a P8 coarse filter, followed by vacuum filtration with a 0.45 μm filter (PALL Life Sciences, VWR, Mississauga ON, CA Cat No. 28148–028). The water extract was frozen at − 80 °C, freeze dried using a lyophilizer and then reconstituted in ddH_2_O in order to obtain a final stock concentration of 100 mg/mL. Prior to use, the water extract was passed through a 0.22 μm filter (Sarstedt, Montreal, QC, CA Cat No. 83.1826.001) in a biosafety cabinet.

### Cell culture

The breast cancer cell line MCF-7 (ATCC® HTB-22™) were cultured in Dulbecco’s Modified Eagle’s Medium (DMEM) (ATCC® 30–2002™) supplemented with 10% (*v*/v) fetal bovine serum (FBS, Thermo Scientific, Waltham, MA, USA, Cat No. 12484–020) and 0.4% (*v*/v) gentamicin (Gibco BRL, VWR, Mississauga, ON, CA Cat No. 15710–064).

The breast cancer cell line MDA-MB-231 (ATCC® HTB-26™) were cultured in Eagle’s Minimum Essential Medium (EMEM) (ATCC® 30–2003™) supplemented with 10% (v/v) fetal bovine serum (FBS) and 0.4% (v/v) gentamicin.

The normal human skin fibroblast cell line (NHF; Coriell Institute for Medical Research, Cat. No. AG09309, Camden, NJ, USA) were cultured in Eagle’s Minimum Essential Medium (EMEM) (ATCC® 30–2003™) supplemented with 10% (v/v) fetal bovine serum (FBS) and 0.4% (v/v) gentamicin.

All cells were maintained in an incubator at 37 °C with 5% CO_2_ and 95% humidity. All cells were cultured for less than 6 months with regular passaging.

### Analysis of cell death: annexin V binding assay and propidium iodide

Annexin V binding assay and propidium iodide staining were performed to respectively monitor early apoptosis and cell permeabilization, a marker of necrotic or late apoptotic cell death. Cells were treated with various concentrations of hibiscus flower extract similar to those published previously with aqueous extracts of dandelion root and white tea [18, 19]. Cells were then treated individually or in combination with chemotherapeutics taxol, cisplatin, and tamoxifen as indicated in the results section. This protocol is similar to that previously published [18, 19]. Cells were washed with phosphate-buffered saline (PBS) and suspended in Annexin V binding buffer (10 mM HEPES, 140 mM NaCl, 2.5 mM CaCl_2_, pH 7.4) with green fluorescent Annexin V AlexaFluor-488 (1:20) (Life Technologies Inc., Burlington, ON, CA, Cat No. A13201) and 0.01 mg/mL of red fluorescent PI (Life Technologies Inc., Burlington, ON, CA, Cat No. P3566) for 15 min at 37 °C protected from light. Percentage of early (green), late apoptotic cells (green and red), and necrotic cells (red) were quantified with a Tali Image-Based Cytometer (Life Technologies Inc., Burlington, ON, CA, Cat No. T10796). Cells from at least 18 random fields were analyzed using both the green (ex. 458 nm; em. 525/20 nm) and red (ex. 530 nm; em. 585 nm) channels. Fluorescent micrographs were taken at 400x magnification using LAS AF6000 software with a Leica DMI6000 fluorescent microscope (Wetzlar, Germany). Cells monitored with microscopy were counterstained with Hoechst 33342 (Molecular Probes, Eugene, OR, USA) with a final concentration of 10 μM during the 15-min incubation.

### Reactive oxygen species (ROS) quantification

Whole cell ROS generation was monitored with the small molecule 2′, 7′-dicholorofluorescin diacetate (H_2_DCFDA). H_2_DCFDA enters the cell and is deacetylated by esterases and oxidized by ROS to the highly fluorescent 2′, 7′-dicholorofluorescein (DCF) (excitation 495 nm; emission 529 nm). This protocol is similar to that previously published [18, 19]. Cells were pretreated with 20 μM H_2_DCFDA (Sigma-Aldrich Canada, Cat. No. D6883, Mississauga, ON, Canada) for 30 min at 37 °C protected from light at 5% CO_2_. Cells were treated for the indicated durations, collected, centrifuged at 3500×g for 5 min, and resuspended in PBS. Percentage of DCF positive cells was quantified using the Tali Image-Based Cytometer (Life Technologies Inc., Burlington, ON, CA, Cat No. T10796) using 13 random fields per group with the green channel (excitation 458 nm; emission 525/20 nm). Cells were monitored with microscopy and counterstained with Hoechst 33342. Images were taken with a Leica DMI6000 fluorescent microscope (Wetzlar, Germany) at 400x magnification using LAS AF6000 software.

### Mitochondrial potential monitoring

Tetramethylrhodamine methyl ester (TMRM) (Gibco BRL, VWR, Mississauga, ON, CA, Cat No. 89139–392) was used for detecting mitochondrial membrane potential (MMP), an indicator of healthy intact mitochondria. This protocol is similar to that previously published [18, 19]. Following incubation with TMRM, cells were collected, washed with 1x PBS, resuspended in PBS, and then analyzed using the Tali Image-Based Cytometer (Life Technologies Inc., Burlington, ON, CA, Cat No. T10796). Cells from 13 random fields were analyzed using the red (ex. 530 nm; em. 585 nm) channel. Cells were monitored with microscopy and counterstained with Hoechst 33342. Images were taken with a Leica DMI6000 fluorescent microscope (Wetzlar, Germany) at 400x magnification using LAS AF6000 software.

### Statistical analysis

All statistical analysis was done using the GraphPad 6.0 Prism software. To test for statistical significance a two-way analysis of variance (ANOVA) was used. All trials were conducted at least three independent times.

## Results

### Hibiscus extract induces apoptosis in a dosage dependent manner in triple-negative and estrogen-receptor positive breast cancer cells

Hot water extract of hibiscus flower was prepared as described in the material and methods. To assess the ability of HE to induce apoptosis in breast cancer, triple-negative and ER+ breast cancer cells were fluorescently stained with apoptosis markers Annexin V (AV) and propidium iodide (PI). The cells were subjected to fluorescent image-based cytometry and fluorescent microscopy following 48- and 96-h treatments.

**Fig. 1:**
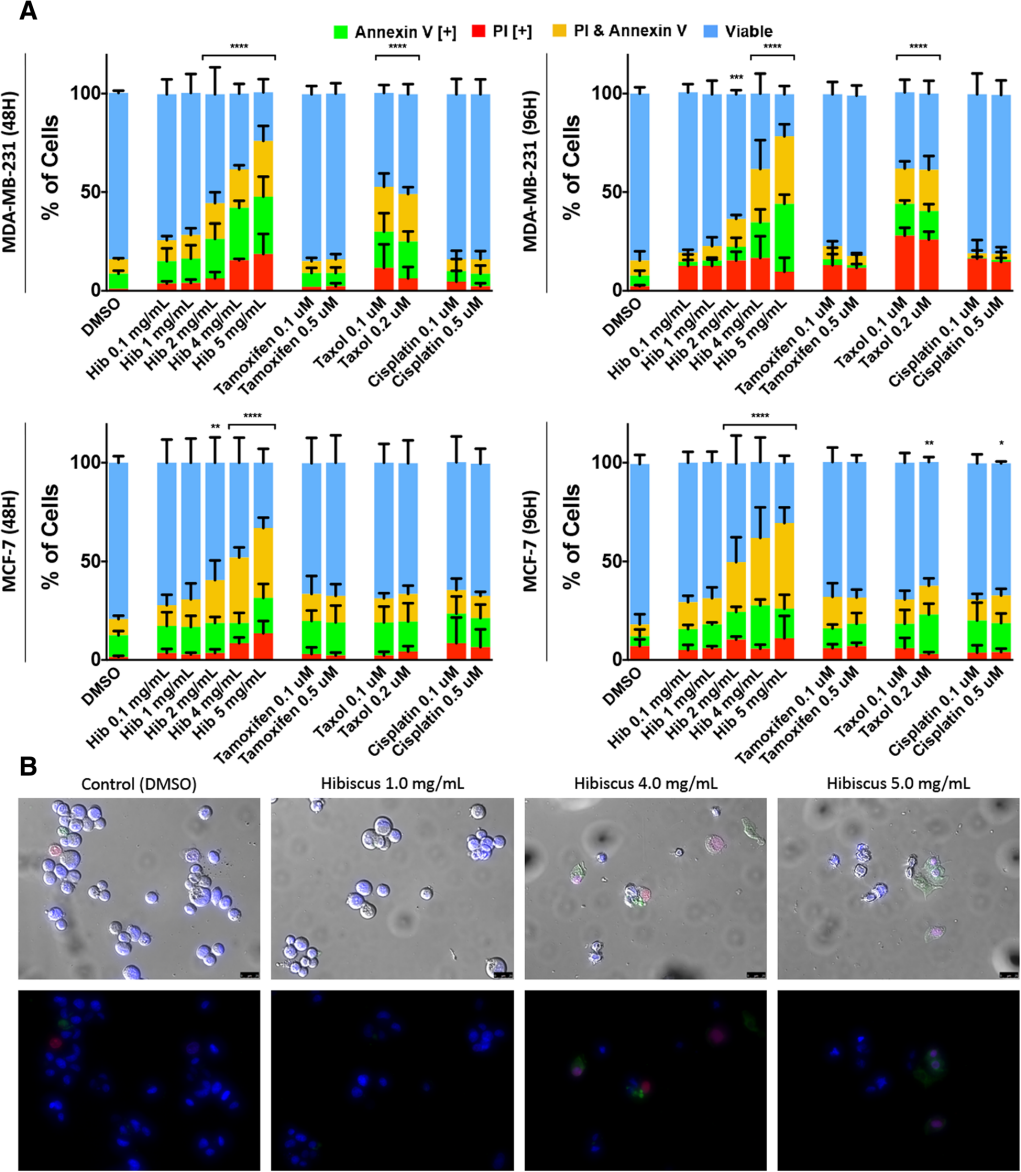
Hibiscus extracts induce apoptosis in breast cancer. **a** Breast cancer cell lines MDA-MB-231 and MCF-7 were treated with various treatments of HE and chemotherapeutics and assessed at 48 h and 96 h. Results were obtained using image-based cytometry to assess the percentage of cells positive with fluorescence associated with Annexin V (green), PI (red), both (yellow), or negative for both Annexin V and PI (blue). Values are expressed as a mean ± SD from three independent experiments. **b** Fluorescence microscopy images of 1.0, 4.0 and 5.0 mg/mL HE treatment on MDA-MB-231 cells were taken at 48 h. Top panels: Brightfield and fluorescent merged images at 400x magnification. Bottom: Fluorescent images stained with Annexin V (green), PI (red), and Hoechst (blue) at 400× magnification. Scale bar is 50 μm. Images are representative of three independent experiments. Statistical calculations were performed using Two-Way ANOVA multiple comparison. *p < 0.05 vs. Control, **p < 0.01 vs. Control, ****p < 0.0001 vs. Control

HE was effective in inducing apoptosis in both triple-negative MDA-MB-231 and ER+ MCF-7 breast cancer cells (Fig. 1a). Specifically, significant apoptosis induction was observed in both breast cancer cell lines at a dosage of 2 mg/mL (2 mg of crude lyophilized extract in 1 mL of ddH_2_O). Dosage dependent apoptosis induction was observed in both cell lines as increasing treatment concentration increased the amount of apoptosis observed.

Both MDA-MB-231 and MCF-7 cells were additionally treated with tamoxifen, taxol, and cisplatin to compare the induction of apoptosis between standard chemotherapeutic treatments and HE. In both cell lines, tamoxifen and cisplatin treatments did not significantly induce apoptosis and taxol significantly induced apoptosis only in MDA-MB-231 cells (Fig. 1a). HE treatment at 4 mg/mL caused significant induction of apoptosis at a comparable or greater level to all chemotherapeutics tested.

Morphological assays were conducted to assess the effect of treatments on cell morphology. Fluorescent microscopy using AV and PI after hibiscus treatments at 48 h confirmed apoptosis induction due to hibiscus. These apoptosis markers were observed in MDA-MB-231 breast cancer cells as expected, along with apoptotic morphology including cell shrinkage, membrane blebbing, and nuclear condensation (Fig. 1b).

### Interaction of hibiscus extract with conventional chemotherapies tamoxifen, taxol, and cisplatin in combination treatments

Commonly today, many chemotherapeutics are utilized in conjunction with other drugs. In order to assess if HE can be combined with chemotherapeutics in a novel treatment regimen, combination assays were conducted to determine whether or not hibiscus enhances, inhibits, or has no effect on chemotherapeutic potency. MDA-MB-231 and MCF-7 breast cancer cells were treated with tamoxifen, taxol, and cisplatin in the presence or absence 1 mg/mL HE. As described above, both image-based cytometry and fluorescent microscopy were used to analyze apoptosis induction.

**Fig. 2:**
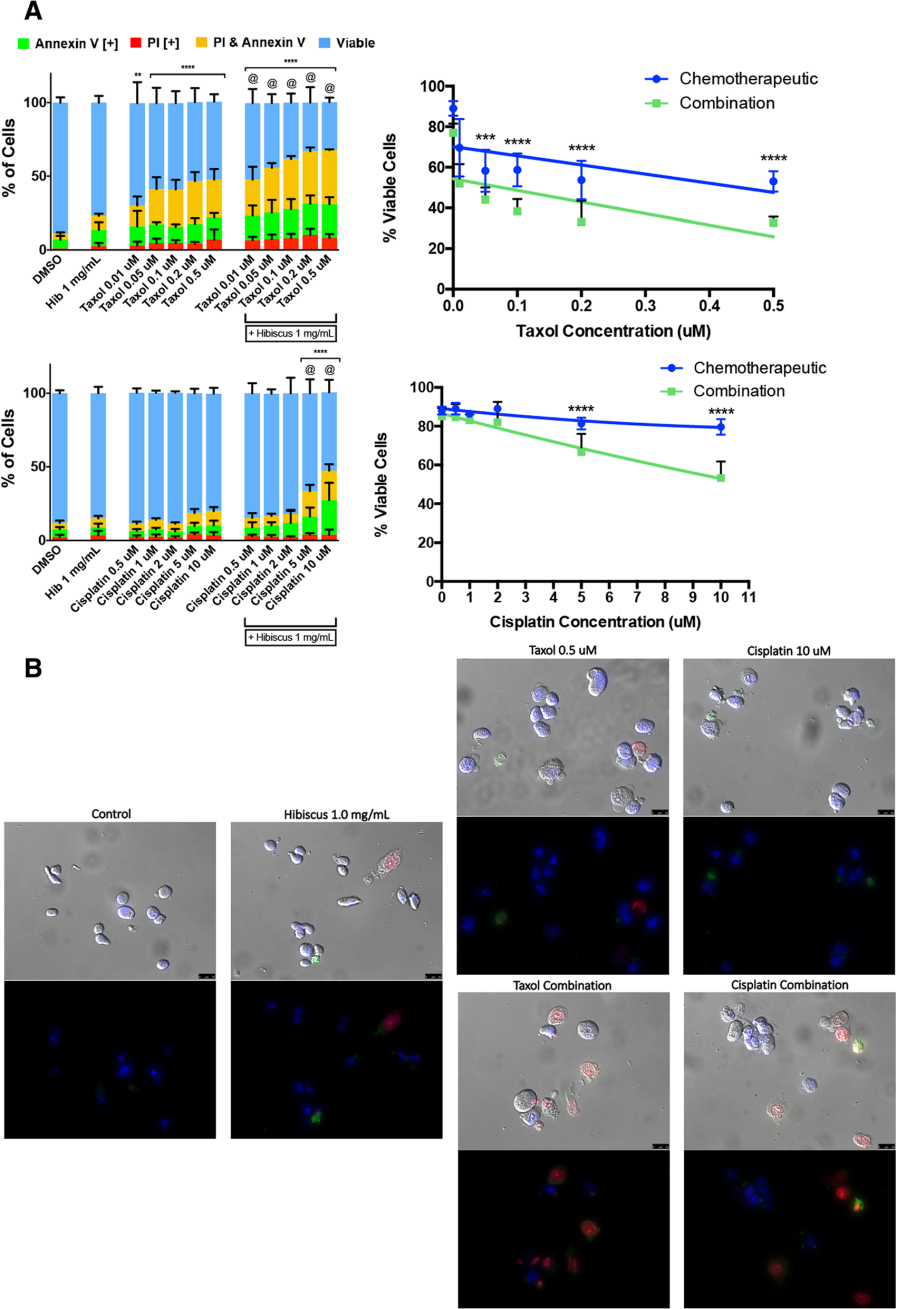
Hibiscus extracts indicate synergy with chemotherapeutics when treated in combination on triple-negative breast cancer cells. **a** MDA-MB-231 cells were treated with chemotherapeutics taxol (top panel) and cisplatin (bottom panel) individually and in combination with 1 mg/mL HE and assessed at 48 h. Results were obtained using image-based cytometry to assess the percentage of cells positive with fluorescence associated with Annexin V (green), PI (red), both (yellow), or negative for both Annexin V and PI (blue). Values are expressed as a mean ± SD from three independent experiments. The percentage of viable cells were graphed for both individual chemotherapeutic and combination chemotherapeutic treatments (graphs on right). **b** Fluorescence microscopy images of individual and hibiscus combination chemotherapeutic treatments on MDA-MB-231 cells were taken at 48 h.. Top panels: Brightfield and fluorescent merged images at 400x magnification. Bottom: Fluorescent images stained with Annexin V (green), PI (red), and Hoechst (blue) at 400× magnification. Scale bar is 50 μm. Images are representative of three independent experiments. Statistical calculations were performed using Two-Way ANOVA multiple comparison. *p < 0.05 vs. Control, **p < 0.01 vs. Control, ****p < 0.0001 vs. Control, @*p* < 0.05 vs. Individual Chemotherapy Treatment

In the triple-negative breast cancer cell line, MDA-MB-231, combination treatments of chemotherapeutics taxol and cisplatin with 1 mg/mL HE were able to significantly increase the induction of apoptosis when compared to chemotherapeutic treatments alone (Fig. 2a). Interestingly, the lowest combination concentration of taxol treatment (0.01 μM with 1 mg/mL HE) showed similar apoptosis induction to the highest individual treatment concentration of taxol (0.5 μM). This indicates that combination treatment with 1 mg/mL HE was able to show a similar apoptosis induction to individual treatment with a 50-fold decrease in chemotherapeutic concentration. Using fluorescent microscopy, this result was confirmed with combination treatments of taxol and cisplatin along with HE showing a higher incidence of apoptotic marker fluorescence and increased apoptotic morphology when compared to individual chemotherapeutic treatments (Fig. 2b).

**Fig. 3:**
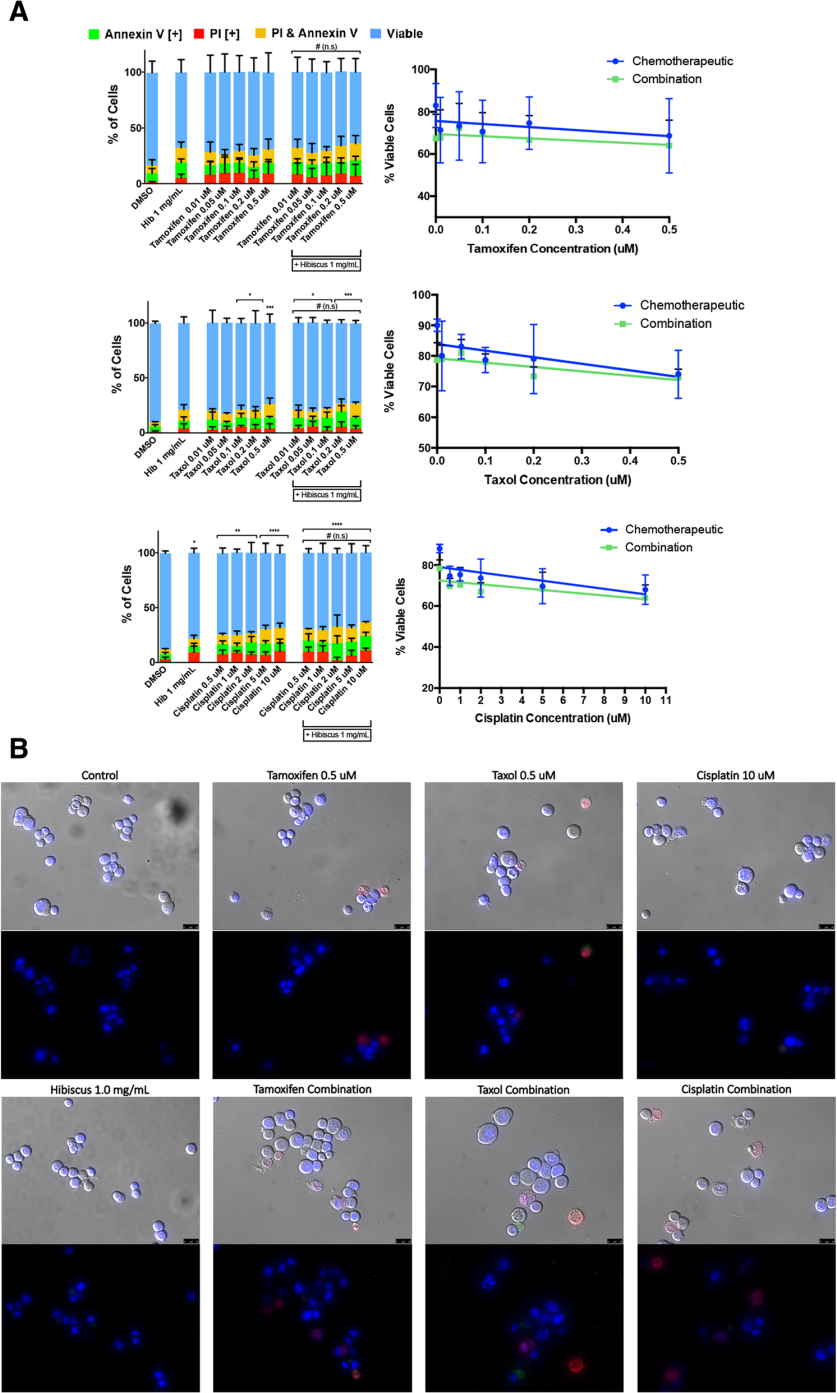
Hibiscus extracts do not interact with chemotherapeutics in combination treatment on estrogen-receptor positive breast cancer. a MCF-7 cells were treated with chemotherapeutics tamoxifen (top panel, taxol (middle panel), and cisplatin (bottom panel) individually and in combination with 1 mg/mL HE and assessed at 48 h. Results were obtained using image-based cytometry to assess the percentage of cells positive with fluorescence associated with Annexin V (green), PI (red), both (yellow), or negative for both Annexin V and PI (blue). Values are expressed as a mean ± SD from three independent experiments. The percentage of viable cells were graphed for both individual chemotherapeutic and combination chemotherapeutic treatments (graphs on right). **b** Fluorescence microscopy images of individual and hibiscus combination chemotherapeutic treatments on MDA-MB-231 cells were taken at 48 h.. Top panels: Brightfield and fluorescent merged images at 400x magnification. Bottom: Fluorescent images stained with Annexin V (green), PI (red), and Hoechst (blue) at 400× magnification. Scale bar is 50 μm. Images are representative of three independent experiments. Statistical calculations were performed using Two-Way ANOVA multiple comparison. *p < 0.05 vs. Control, **p < 0.01 vs. Control, ****p < 0.0001 vs. Control, @*p* < 0.05 vs. Individual Chemotherapy Treatment, # = not significant vs. Individual Chemotherapy Treatment

In ER+ breast cancer cell line, MCF-7, combination treatments of chemotherapeutics tamoxifen, taxol and cisplatin with 1 mg/mL HE did not show any significant change in apoptosis induction when compared to individual treatments (Fig. 3a). Although we did not observe any enhancement, there was no inhibition observed. This result was confirmed using fluorescent microscopy (Fig. 3b). However, it is important to note that the chemotherapeutic treatment ranges used did not show any significant apoptosis induction in MCF-7. As shown in Fig. 1, HE at a concentration of 2 mg/mL showed significant apoptosis induction while combination treatments with 1 mg/mL did not induce significant apoptosis.

### Hibiscus extract is selective in inducing apoptosis for breast cancer cells

**Fig. 4:**
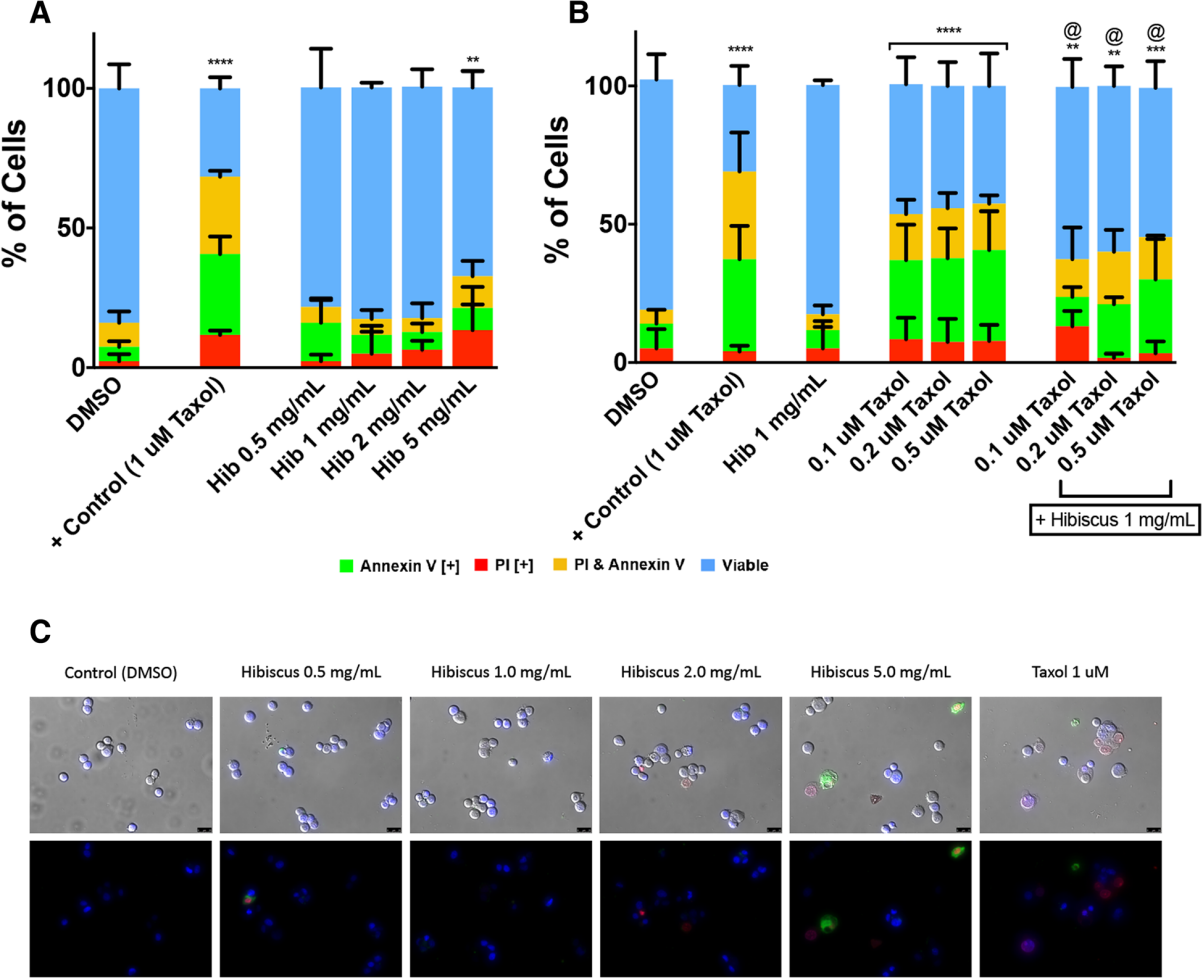
Hibiscus extracts are selective for cancer and reduce toxicity of chemotherapeutics. **a** NHF cells were treated with various dosages of HE and (**b**) hibiscus combination treatments with taxol and assessed at 48 h. Results were obtained using image-based cytometry to assess the percentage of cells positive with fluorescence associated with Annexin V (green), PI (red), both (yellow), or negative for both Annexin V and PI (blue). Values are expressed as a mean ± SD from three independent experiments. The percentage of viable cells were graphed for both individual chemotherapeutic and combination chemotherapeutic treatments (graphs on right). **c** Fluorescence microscopy images of individual hibiscus treatments on NHF cells were taken at 48 h. Top panels: Brightfield and fluorescent merged images at 400x magnification. Bottom: Fluorescent images stained with Annexin V (green), PI (red), and Hoechst (blue) at 400× magnification. Scale bar is 50 μm. Images are representative of three independent experiments. Statistical calculations were performed using Two-Way ANOVA multiple comparison. *p < 0.05 vs. Control, **p < 0.01 vs. Control, ****p < 0.0001 vs. Control, @*p* < 0.05 vs. Individual Chemotherapy Treatment

If selective for breast cancer, individual and combinatory HE treatment could potentially minimize adverse side effects by not affecting healthy cells. In order to investigate the selectivity of HE for breast cancer, normal human fibroblast (NHF) cells were treated with HE treatments and assessed in a similar manner as described above. Compared to control treatments, there was no increase in apoptosis in HE up to 2 mg/mL at which we have observed significant apoptosis in cancer cells (Fig. 1). There was minimal to no observable apoptosis induction when compared to the positive control taxol (at a high dosage known to be cytotoxic to normal human cells) using HE treatments that were highly efficacious when used to treat breast cancer cells (Fig. 4a). These results were confirmed with fluorescent microscopy. Cells only began to show apoptotic marker fluorescence and apoptotic morphology at the highest HE concentration of 5 mg/mL (Fig. 4c).

To further investigate the benefit of using a combination treatment of HE with chemotherapeutics, taxol and hibiscus combination treatments were compared to individual treatments of taxol on NHF cells. On their own, chemotherapeutics treatments showed toxicity (Fig. 4b). They are non-selective compared to hibiscus. Most surprisingly, HE combination treatments did not lead to increased apoptosis induction when compared to individual treatments, but instead lowered the amount of apoptosis induction observed (Fig. 4b). These results indicate that HE shows selectivity to breast cancer cells and potentially protects normal human healthy cells from being affected by chemotherapeutic treatments.

### Hibiscus extract is able to induce apoptosis in breast cancer cells by increasing oxidative stress and targeting the mitochondria

**Fig. 5:**
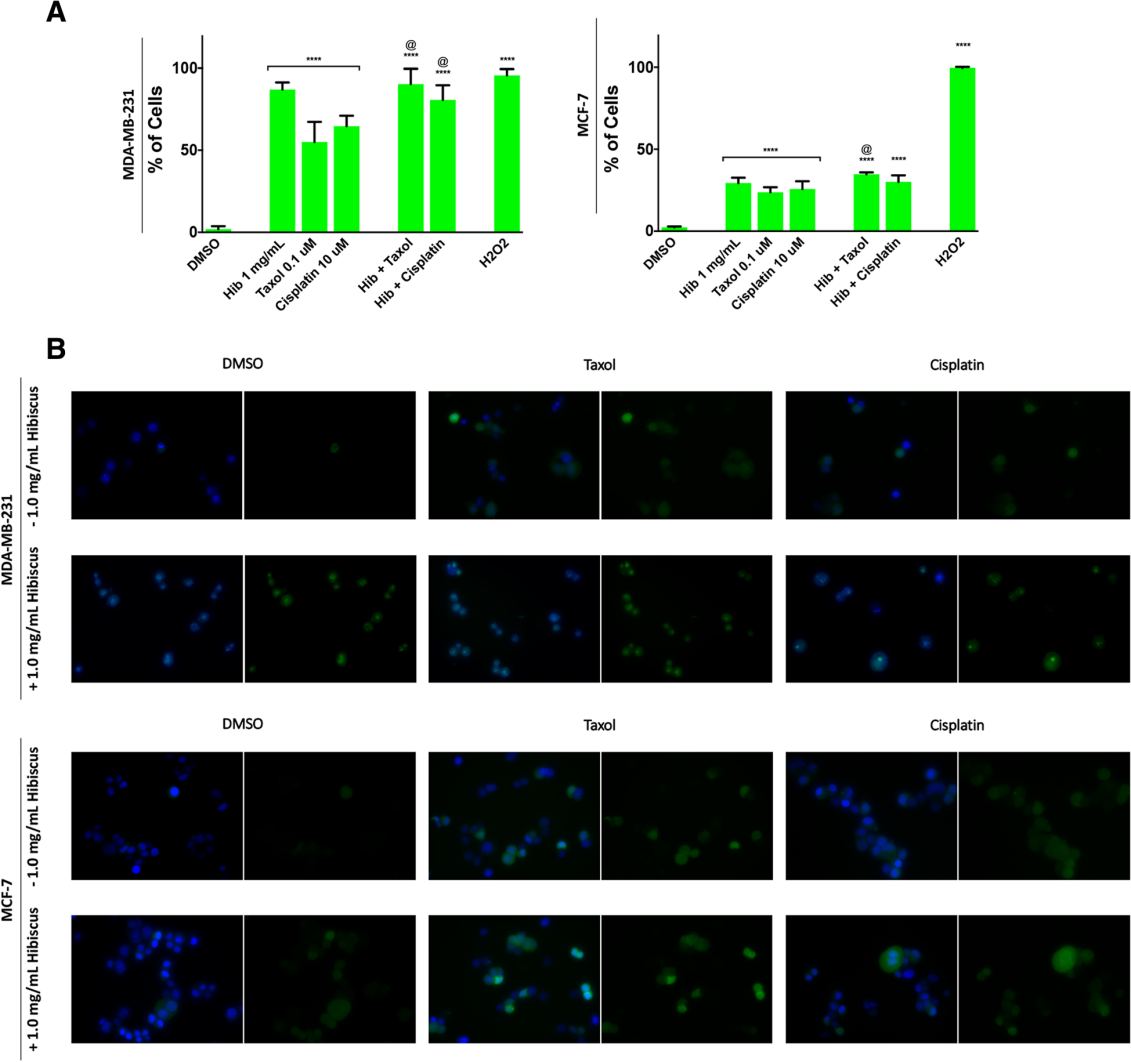
Hibiscus extract induces oxidative stress on breast cancer and enhances chemotherapeutic oxidative stress induction. **a** MDA-MB-231 (left) MCF-7 (right) breast cancer cells were treated with chemotherapeutics taxol and cisplatin individually and in combination with 1 mg/mL HE and assessed at 3 h post-treatment against a positive control of hydrogen peroxide (H_2_O_2_). Results were obtained using image-based cytometry to assess the percentage of cells positive with fluorescence associated with the generation of reactive oxygen species (H_2_DCFDA, fluoresces green). Values are expressed as a mean ± SD from three independent experiments. **b** Fluorescence microscopy images of individual and hibiscus combination chemotherapeutic treatments on MDA-MB-231 and MCF-7 cells were taken at 3 h.. Left image in groupings: Fluorescent images stained with H_2_DCFDA (green) and Hoechst (blue) at 400× magnification. Right image in groupings: Fluorescent images stained with H_2_DCFDA (green) alone. Images are representative of three independent experiments. Statistical calculations were performed using Two-Way ANOVA multiple comparison. *p < 0.05 vs. Control, **p < 0.01 vs. Control, ****p < 0.0001 vs. Control, @*p* < 0.05 vs. Individual Chemotherapy Treatment

HE is an extract composed of many compounds able to interact in a complex manner. Determining the method of apoptosis induction will allow for a greater understanding of how these complex extracts show the observed anticancer potency. In order to determine if HE is able to induce apoptosis in breast cancer through inducing oxidative stress, H_2_DCFDA was used to monitor the generation of ROS in breast cancer cells treated with chemotherapeutics in the presence or absence of HE. Indeed, it was observed that individual HE treatment was able to induce significant ROS generation in treated cells (Fig. 5a). Further, combination treatments on triple-negative MDA-MB-231 cells using chemotherapeutic and HE were able to significantly increase the generation of ROS in treated cells when compared to treatment in the absence of HE. These results were confirmed using fluorescence microscopy (Fig. 5b).

**Fig. 6:**
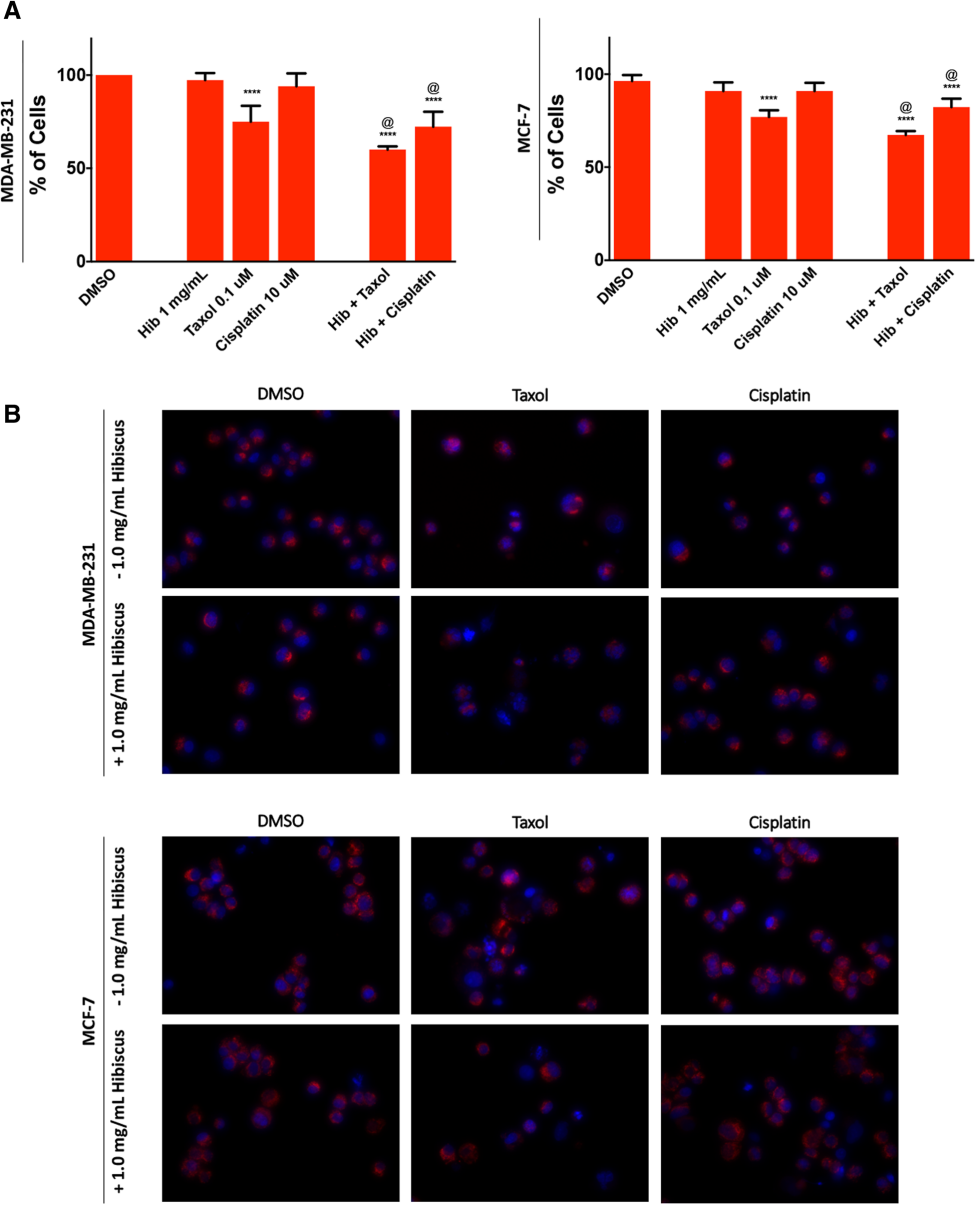
Hibiscus extract enhances chemotherapeutic ability to reduce mitochondrial membrane potential. **a** MDA-MB-231 (left) MCF-7 (right) breast cancer cells were treated with chemotherapeutics taxol and cisplatin individually and in combination with 1 mg/mL HE and assessed at 48 h. Results were obtained using image-based cytometry to assess the percentage of cells positive with fluorescence associated with mitochondrial membrane potential (TMRM, fluoresces red). Values are expressed as a mean ± SD from three independent experiments. **b** Fluorescence microscopy images of individual and hibiscus combination chemotherapeutic treatments on MDA-MB-231 and MCF-7 cells were taken at 48 h.. Fluorescent images stained with TMRM (red) and Hoechst (blue) at 400× magnification. Images are representative of three independent experiments. Statistical calculations were performed using Two-Way ANOVA multiple comparison. *p < 0.05 vs. Control, **p < 0.01 vs. Control, ****p < 0.0001 vs. Control, @*p* < 0.05 vs. Individual Chemotherapy Treatment

Further, as HE is made up of multiple factors and components, some of these may also target the mitochondria. Tetramethylrhodamine methyl ester (TMRM) dye was used in order to visualize the mitochondria membrane potential (MMP) in treated cells. Interestingly, HE at 1 mg/mL did not show significant loss of the MMP but instead was able to amplify the loss of MMP in both triple-negative and ER+ breast cancer cells when present in chemotherapeutic treatment compared to when absent (Fig. 6a). These results were confirmed using fluorescent microscopy (Fig. 6b).

## Discussion

In this study, we have shown that HE is able to induce apoptosis in both human ER+ and triple-negative breast cancer cell lines in vitro (Fig. 2a). We have demonstrated that HE treatment is very selective in inducing cell death in cancer cells without any significant effect on NHF cells (Fig. 4a). On the other hand, common chemotherapeutics like taxol were indiscriminate and induced apoptosis in both cancer and non-cancerous cells (Fig. 4a). Most importantly, we have shown that addition of HE in combination with chemotherapeutic agents enhanced the induction of apoptosis in triple-negative breast cancer cells (Fig. 2a). These results support the possibility of supplementing chemotherapeutic regimens with HE, which is well-tolerated in normal healthy cells. This may lead to a better combined effect, reducing the chemotherapeutic dosages needed in treatment and therefore reduce toxicity.

As indicated previously, breast cancer, primarily triple-negative, is highly resistant to chemotherapy treatment. We have shown that both triple-negative and ER-positive breast cancer cells are affected by HE treatment (Fig. 2). HE has also been shown to induce apoptosis significantly around treatment of 2 mg/mL of crude extract in prostate cancer, with a similar dose-dependency [24].

A common hesitation of using natural health product extracts alongside chemotherapies is the possibility of negative drug interactions, leading to reduced efficacy in treatment. Our goal was to investigate whether or not combination HE treatments would inhibit, not affect, or enhance the efficacy of chemotherapeutic treatments. Indeed, we found that taxol and cisplatin treatments on triple-negative breast cancer cells were enhanced with the addition of 1 mg/mL (sublethal dosage in individual treatment) HE treatment (Fig. 2) while unaffected in ER-positive breast cancer cells. These results clearly indicate that HE’s interaction with chemotherapeutic drugs is positive or has no interaction in breast cancer cells. If any effect was observed at all, HE treatment enhanced the efficacy of chemotherapeutic treatments. Moreover, HE combination treatments on NHF cells were able to reduce the toxicity of taxol (Fig. 4a). Extent of apoptosis induced by 0.01 μM taxol in combination with HE was equivalent to that induced by 0.5 μM taxol alone (Fig. 2a). This 50-fold decrease in effective chemotherapy concentration clearly indicates the possibility of reducing chemotherapeutic dosage to avoid adverse side-effects without sacrificing efficacy. As such, HE could serve a significant purpose in terms of adjuvant therapy.

The mechanism of apoptosis induction in breast cancer is a topic of great interest to determine the underlying cause of cell death. Previously, we have shown that ethanolic extracts of lemongrass and aqueous extracts of dandelion root were able to induce oxidative stress and decrease mitochondrial membrane potential, leading to apoptosis induction in cancer cells [18, 19]. While the exact mechanism is not yet clear, it has been hypothesized that high ROS levels can activate cellular stress mechanisms and may sensitize cancer cells to further ROS production leading to apoptosis [9]. Indeed, our results indicate that HE treatment led to increased ROS generation in both triple-negative and ER+ breast cancer cells (Fig. 5). Moreover, taxol and cisplatin treatments in combination with HE showed increased ROS generation when compared to individual treatments. This helps explain the increase in apoptosis induction of combination treatments compared to individual treatments as discussed above (Figs. 3, 4). It should be noted that triple-negative breast cancer cells were more vulnerable to oxidative stress than ER-positive breast cancer cells. These are two different cells with varied susceptibilities, and the lowered ROS generation of ER-positive breast cancer treatment indicates either an alternate mechanism of apoptosis induction or a need for increased dosage. Further, we have demonstrated that HE combination treatment is able to enhance the mitochondrial membrane potential reduction in breast cancer cells (Fig. 6).

As indicated above, cisplatin and taxol have shown extremely toxic side effects due to a lack of selectivity in treatment. Studies have indicated that HE is well-tolerated in nude mice xenograft models while exhibiting an anti-metastatic and anti-tumour effect [24]. Hibiscus has been traditionally used and has shown to be well-tolerated when consumed by humans. Consumption has also been associated with many beneficial effects including supporting mitochondrial function, energy homeostasis and improvement of the cardiovascular health [32]. Indeed, we have shown that HE was selectively toxic to cancer cells wherein the lowest effective dose of HE on breast cancer (2 mg/mL) was unable affect NHF cells (Fig. 4a). HE treatments in combination with chemotherapeutics were also able to reduce the toxicity in NHF cells and lower the amount of apoptosis induction when compared to chemotherapeutic treatments in the absence of HE (Fig. 4b). As such, HE shows great potential as an adjuvant to these therapies and help render some selectivity in treatment for cancer. If HE treatment shows anticancer efficacy, it could be used over a long-term period of time without any side effects [33].

It is important to note that HE dosages may appear to be high compared to pure compound cancer therapeutics. However, it is important to note that this is an aqueous extract of the hibiscus flower, which contains mainly sugars, salts, and other naturally abundant compounds in flowers. Previous work on phytochemical analysis of many other extracts including long pepper (*Piper longum*) and dandelion root (*Taraxacum officinale*) have shown that the concentration of the active compound might be very low [18, 20]. Further, our work on these NHPs showed that active compounds found in long pepper and dandelion root extract were ineffective in apoptosis induction when used alone [20]. This indicates the importance of multiple phytochemicals that work together natural extracts. In this case, it represents a very interesting opportunity for further research into HE to identify and test the potency of active compounds in aqueous hibiscus flower extract.

## Conclusions

The work presented in this study indicates great potential of NHPs such HE to treat breast cancer in combination with standard chemotherapies. HE has shown an ability to enhance apoptotic induction by chemotherapy treatments through an increase in ROS generation and mitochondrial membrane collapse on both triple-negative and ER-positive breast cancer cells. This result is significant due to the general difficulty in discovery of an effective treatment for resistant triple-negative breast cancer. Most importantly, addition of HE with chemotherapeutic treatment could produce desired level of apoptotic induction at very low dosages of chemotherapies compared to chemotherapies alone. Therefore, addition of HE can significantly reduce the drug-related toxicity of chemotherapeutics. Future work into assessment of HE can look into combinatorial effects on in vivo models to further investigate the potential of HE for human usage. We have shown that HE treatment has the potential to be used alongside tamoxifen, taxol, and cisplatin treatments without any inhibition of drug potency. Thus, these findings open up interesting opportunity for further development of NHPs as a promising anticancer treatment option.

## Abbreviations

AV: Annexin V
DCF: 2′, 7′-dicholorofluorescein
ddH_2_O: Double distilled water
DMSO: Dimethyl sulfoxide
ER+: Estrogen-receptor positive
H_2_DCFDA: 2′, 7′-dicholorofluorescin diacetate
HE: Hibiscus extract
MMP: Mitochondria membrane potential
NHF: Normal human fibroblast
NHP: Natural health product
PBS: Phosphate buffer saline
PI: Propidium iodide
ROS: Reactive oxygen species
TMRM: Tetramethylrhodamine methyl ester
